# Who is Reached by HIV Self-Testing? Individual Factors Associated With Self-Testing Within a Community-Based Program in Rural Malawi

**DOI:** 10.1097/QAI.0000000000002412

**Published:** 2020-10-01

**Authors:** Pitchaya P. Indravudh, Bernadette Hensen, Rebecca Nzawa, Richard Chilongosi, Rose Nyirenda, Cheryl C. Johnson, Karin Hatzold, Katherine Fielding, Elizabeth L. Corbett, Melissa Neuman

**Affiliations:** a Department of Global Health and Development, London School of Hygiene and Tropical Medicine, London, United Kingdom; b Malawi-Liverpool-Wellcome Trust Clinical Research Program, Blantyre, Malawi; c Department of Clinical Research, London School of Hygiene and Tropical Medicine, London, United Kingdom; d Population Services International, Lilongwe, Malawi; e Department of HIV and AIDS, Ministry of Health, Lilongwe, Malawi; f Global HIV, hepatitis and sexually transmitted infections programs, World Health Organization, Geneva, Switzerland; g Population Services International, Washington D.C; h Department of Infectious Disease Epidemiology and MRC Tropical Epidemiology Group, London School of Hygiene and Tropical Medicine, London, United Kingdom

**Keywords:** HIV self-testing, HIV testing, Malawi, men, population-based survey, causal associations

## Abstract

**Introduction:**

HIV self-testing (HIVST) is an alternative strategy for reaching population subgroups underserved by available HIV testing services. We assessed individual factors associated with ever HIVST within a community-based program.

**Setting:**

Malawi.

**Methods:**

We conducted secondary analysis of an end line survey administered under a cluster-randomized trial of community-based distribution of HIVST kits. We estimated prevalence differences and prevalence ratios (PRs) stratified by sex for the outcome: self-reported ever HIVST.

**Results:**

Prevalence of ever HIVST was 45.0% (475/1055) among men and 40.1% (584/1456) among women. Age was associated with ever HIVST in both men and women, with evidence of a strong declining trend across categories of age. Compared with adults aged 25–39 years, HIVST was lowest among adults aged 40 years and older for both men [34.4%, 121/352; PR 0.74, 95% confidence interval (CI): 0.62 to 0.88] and women (30.0%, 136/454; PR 0.71, 95% CI: 0.6 to 0.84). Women who were married, had children, had higher levels of education, or were wealthier were more likely to self-test. Men who had condomless sex in the past 3 months (47.9%, 279/582) reported a higher HIVST prevalence compared with men who did not have recent condomless sex (43.1%, 94/218; adjusted PR 1.37, 95% CI: 1.06 to 1.76). Among men and women, the level of previous exposure to HIV testing and household HIVST uptake was associated with HIVST.

**Conclusions:**

Community-based HIVST reached men, younger age groups, and some at-risk individuals. HIVST was lowest among older adults and individuals with less previous exposure to HIV testing, suggesting the presence of ongoing barriers to HIV testing.

## Introduction

Early diagnosis of people living with HIV (PLHIV) is critical to prevent new HIV infections. Knowledge of HIV status among PLHIV has rapidly increased in sub-Saharan Africa over the past decade.^1^ In 2017, 73% of PLHIV in Malawi knew their HIV status, of whom 90% were on treatment, of whom 91% were virally suppressed.^2^ However, the proportion of PLHIV aware of their HIV status is lower among men than women, with 68% of HIV-positive men diagnosed compared with 76% of HIV-positive women.^2^ Relative to older adults, adolescents and young adults aged 16–24 years also have poor knowledge of their HIV-positive status, increasing their risk of transmission and delay of treatment.^2,3^

Most HIV testing services (HTS) in Malawi are provided at health facilities, although sex and age-specific barriers to facility-based HTS continue to exist.^4^ Men have lower rates of health care utilization in general, reducing their opportunities to test for HIV through routine services.^5^ Masculine norms might also lead men to underestimate HIV risk or symptoms of illness, prioritize economic obligations, stigmatize use of HTS, or fear knowing their HIV status.^5–8^ For adolescents or young adults, their status as dependents can limit their ability to consent or pay for HTS or generate fears of social and economic marginalization from families after an HIV-positive diagnosis.^9–11^ Concerns around revealing sexual debut or stigma and discrimination from health care providers can also limit access to HTS.^9,10^

HIV self-testing (HIVST) is an alternative strategy for reaching population subgroups underserved by available HTS. In 2016, HIVST was recommended by the WHO based on evidence of high acceptability, feasibility, accuracy, and uptake.^12^ Randomized trials in sub-Saharan Africa have demonstrated the effectiveness of HIVST on increasing HIV testing coverage in men and adolescents.^13–18^ The appeal of HIVST is that individuals are able to learn their HIV status in a convenient and discreet manner while achieving greater control and empowerment over the HIV testing process.^12,19^

Providing HTS through alternative approaches is important to meet global treatment and prevention goals. Determining the characteristics of individuals who self-test for HIV is essential to understanding the added value of HIVST programs in closing gaps in HIV testing coverage. Few population-based studies have examined characteristics of individuals who are accessing HIVST programs in sub-Saharan Africa. Here, we assessed individual factors associated with self-reported ever HIVST within a community-based program in rural Malawi.

## Methods

### Parent Study: Design, Sampling, and Data Collection

The parent study was a pragmatic cluster-randomized trial evaluating the effectiveness of community-based distribution of HIVST kits on uptake of HIV testing and antiretroviral therapy (ART) initiation (Clinical trial number: NCT02718274).^15^ The trial was delivered in rural Blantyre, Machinga, Mwanza, and Neno districts in southern Malawi, which has an estimated HIV prevalence of 12.8%.^20^ Twenty-two government primary health centers and their catchment areas were enrolled and randomized 1:1 to the community-based HIVST intervention or the standard of care. The study population included residents aged 16 years and older in health facility-defined clusters. Trial outcomes were assessed through population-representative surveys administered to cluster residents at baseline and end line. The trial is described elsewhere in detail.^21^

From September 2016 to January 2018, HIVST kits were delivered through community-based distribution agents (CBDAs), an established cadre of resident volunteers who deliver health commodities in Malawi. Implementation was led by Population Services International Malawi. Trained CBDAs promoted oral fluid-based kits door-to-door, with cluster residents aged 16 years and older eligible for HIVST. Informed consent to take an HIVST kit was waived by research ethics committees. Residents received an explanation on how to use the kit, interpret the results, and access onward HIV care and prevention services. Instructions were supplemented by a demonstration-of-use and instructional materials. CBDAs received a stipend for each kit distributed (MWK 100/USD 0.15). HTS and ART services could be accessed at health facilities as part of the standard of care.

The end line survey to evaluate the impact of the community-based HIVST program was administered from October 2017 to January 2018. The survey used a two-stage sampling design. Evaluation villages meeting defined inclusion criteria were randomly selected per cluster. Households in each evaluation village were enumerated by research assistants and a variable proportion of households were randomly selected for the survey. A sample size of 250 individuals per cluster was calculated based on trial outcomes.

All individuals aged 16 years and older in selected households were eligible for the survey. Research assistants made multiple household visits to schedule interviews with eligible household members. After informed consent or assent, individual-level questionnaires were administered to participants on sociodemographic characteristics, HIV testing and HIVST, and sexual behavior. The head of household or representative also completed a household-level module on socioeconomic status.

### Current Study: Outcome and Exposure Measurement

This study consists of secondary analysis of the end line survey administered in the 11 community-based HIVST intervention clusters. The outcome of interest was self-reported ever HIVST. We decided to use ever HIVST, in contrast to HIVST in the past 12 months, to ensure that HIVST uptake across the 14 to 17-month intervention period was captured, with a limited prevalence (<1%) of HIVST reported at baseline.^15^ Exposures included age group, sociodemographic factors (head of household, married, and children), socioeconomic factors (educational attainment and household wealth status), sexual behavior factors (condomless sex in the past 3 months), and health behavior factors (self-rated health status, number of HIV tests before the past 12 months, and household uptake of HIVST).

A household wealth index was constructed using principal components analysis from an inventory of household and individual assets (see Table 1, Supplemental Digital Content, http://links.lww.com/QAI/B485).^22^ Values were then divided into tertiles. Condomless sex with at least one sexual partner in the past 3 months was derived from a set of 5 questions on sexual behavior and acted as a proxy for sexual risk (see Table 2, Supplemental Digital Content, http://links.lww.com/QAI/B485). Number of HIV tests before the past 12 months was estimated using the difference between the number of lifetime tests and number of tests in the past 12 months. The measure was used to approximate exposure to HIV testing before the community-based HIVST program. A binary measure for household uptake of HIVST was generated based on whether another household member reported ever HIVST.

### Statistical Analysis

Observations with missing age, sociodemographic, or socioeconomic data were excluded from the analysis (Fig. 2). As the outcome was common, we calculated prevalence differences using a binomial regression model and prevalence ratios (PRs) using a Poisson regression model with a robust variance estimator.^23,24^ Data analysis was stratified by sex, with factors associated with HIVST considered likely to vary given known differences in HIV testing coverage between men and women. Clustering was adjusted for using a fixed effect of health facility. P values were obtained using Wald tests.

**Figure 1:**
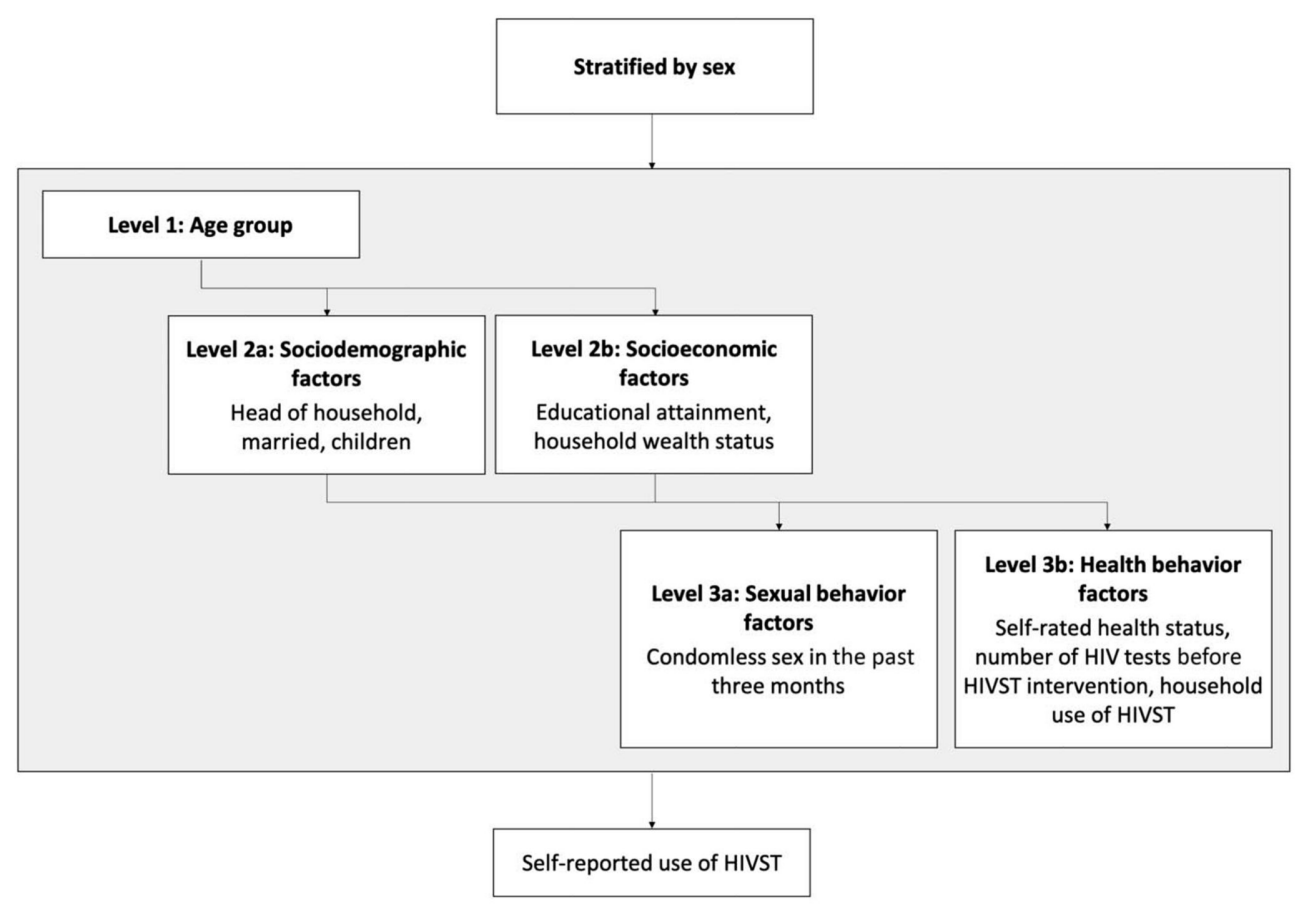
Conceptual framework of causal relationships between exposures and HIV self-testing. Illustration of hypothesized relationships between exposures and HIV self-testing, with more distal covariates considered likely to confound the relationship between more proximal covariates and the outcome.

To test for causal associations between the exposures and outcomes, we purposefully identified and adjusted for con-founders using the conceptual framework in Figure 1.^25^ Covariates were categorized and then ordered based on a hypothesized hierarchy of their relationship with the outcome, with more distal covariates considered likely to confound the relationship between more proximal covariates and the outcome. Effect estimates were then adjusted for covariates higher in the conceptual framework, thereby likely not on the casual pathway, and associated with the outcome (P < 0.10) in the unadjusted analysis. Specifically, models assessing sociodemographic or socioeconomic factors controlled for age. Models assessing sexual or health behavior factors controlled for age, sociodemographic, and socioeconomic variables.

We used multiple imputations for the measure on condomless sex in the past 3 months due to the high proportion of missing observations. Our imputation model included variables believed to predict responses on sexual behavior, including age, sociodemographic variables, socioeconomic variables, cluster, and the outcome.^26,27^ We used 25 imputations based on the proportion of missing cases, and Rubin’s rules to obtain combined estimates from the imputed data.^27^

Data were analyzed in Stata version 14.0.

### Ethics Statement

The parent study received ethical approval from the London School of Hygiene & Tropical Medicine and the University of Malawi College of Medicine. Informed verbal consent for the end line survey was obtained for individuals aged 18 years and older. Individuals aged 16 and 17 years were asked to give verbal assent, with consent obtained from their parents or guardians.

## Results

### Response Rate and Sample Characteristics

**Figure 2:**
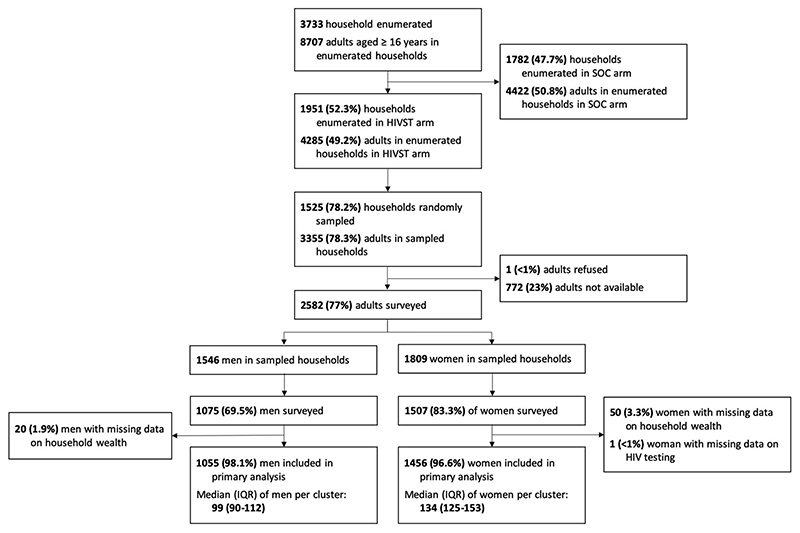
Flow diagram of study participation. Flow diagram of household and individual participation in the end line survey. IQR, interquartile range; SOC, standard of care.

Household enumeration identified 4285 individuals aged 16 years and older, with 3355 individuals eligible for the survey following random household sampling (Fig. 2). Individual-level response rates were 69.5% (1075/1546) for men and 83.3% (1507/1809) for women, with most remaining household members unavailable. Of consenting men and women, 98.1% (1055/1075) and 96.6% (1456/1507) had complete data for age, sociodemographic factors, and socioeconomic factors, respectively.

Sample characteristics for men and women are described in Table 1. Distribution of age group was similar by sex. Relative to women, more men reported being the head of household or married, although fewer reported having children. Men were also more educated and resided in wealthier households than women. A higher proportion of men (72.8%, 582/800) reported having condomless sex in the past 3 months compared with women (56.5%, 608/1077). The proportion of men who had not tested before the past 12 months (28.3%, 287/1055) was also higher than women (19.1%, 260/1456).

### Self-Reported Ever HIV Self-Testing

Prevalence of self-reported ever HIVST was 45.0% (475/1055) among men and 40.1% (584/1456) among women. Cluster-level coverage of HIVST ranged from 26.5% to 69.6% for men and 23.1% to 66.0% for women. Furthermore, 83.4% (880/1055) of men and 89.1% (1298/1456) of women reported ever HIV testing.

A quarter of men (24.2%, n = 255) had incomplete data on condomless sex in the past 3 months, of whom 40.0% (n = 102) reported HIVST compared with 46.6% (373/800) of men with complete data. Among women, 25.6% (n = 379) had missing data, with 42.0% (n = 159) self-testing in the missing group vs. 39.5% (425/1077) self-testing in the nonmissing group.

The results of the multivariable regression models are shown for men in Table 2 and for women in Table 3.

### Age

Age was associated with ever HIVST in both men and women, with evidence of a strong declining trend across categories of age. HIVST was higher among adolescent boys aged 16–19 years (50.7%, 70/138) than men aged 25–39 years (46.9%, 179/382), although the confidence interval included the null value [PR 1.02, 95% confidence interval (CI): 0.84 to 1.22]. A higher proportion of young men aged 20–24 years had also self-tested compared with adolescent boys (57.4%, 105/183; PR 1.2, 95% CI: 1.02 to 1.41). Relative to women aged 25–39 years (41.3%, 217/525), HIVST seemed to be more prevalent in adolescent girls aged 16–19 years (46.4%, 97/209; PR 1.12, 95% CI: 0.93 to 1.33) and young women aged 20–24 years (50.0%, 134/268; PR 1.15, 95% CI: 0.99 to 1.34), although evidence for the effect was weak. HIVST was lowest among adults aged 40 years and older for both men (34.4%, 121/352; PR 0.74, 95% CI: 0.62 to 0.88) and women (30.0%, 136/454; PR 0.71, 95% CI: 0.6 to 0.84).

### Sociodemographic and Socioeconomic Factors

Married women or women living with their partner (43.8%, 424/968) had a higher prevalence of ever HIVST than women who were not married or cohabitating (32.8%, 160/488; adjusted PR 1.26, 95% CI: 1.09 to 1.46). Furthermore, 40.5% (510/1260) of women with children had selftested compared with 37.8% (74/196) of women without children (adjusted PR 1.38, 95% CI: 1.08 to 1.76). Marital and parental status were not associated with HIVST among men. Across sex, there was no evidence that being the head of household was associated with HIVST (Tables 2 and 3).

Higher educational attainment and household wealth status were strongly associated with HIVST in women but not in men. Compared with women with no formal education (30.5%, 102/334), HIVST was more prevalent for women who had completed primary education (41.9%, 409/975; adjusted PR 1.31, 95% CI: 1.09 to 1.57) or secondary education or higher (49.7%, 73/147; adjusted PR 1.66, 95% CI: 1.29 to 2.13). In terms of household wealth status, a higher proportion of women in the highest wealth tertile had self-tested (44.8%, 219/489) relative to women in the lowest wealth tertile (35.7%, 175/490; adjusted PR 1.3, 95% CI: 1.12 to 1.52).

### Sexual and Health Behavior Factors

In the multiple imputation analysis, men who had sexual intercourse without a condom in the past 3 months (47.9%, 279/582) reported a higher prevalence of ever HIVST compared with men who did not have recent condomless sex (43.1%, 94/218; adjusted PR 1.37, 95% CI: 1.06 to 1.76). Among women, there was no evidence of an association between sexual risk and HIVST. Estimates were similar across multiple imputations and complete case analyses (Tables 2 and 3).

Frequent HIV testing before the past 12 months was strongly associated with HIVST in men and women. HIVST was more common among men who had previously tested 1–2 times (47.2%, 143/303) than men who had not tested (23.3%, 67/287; adjusted PR 2.01, 95% CI: 1.59 to 2.54), with increased HIVST based on the previous number of tests. Women who had previously tested 1–2 times (39.2%, 164/418) also had a higher HIVST prevalence compared with women who had not tested (18.1%, 47/260; adjusted PR 2.03, 95% CI: 1.52 to 2.71).

Living with a household member who ever self-tested was associated with HIVST. Among men, 64.4% (322/500) who reported household uptake self-tested compared with 27.6% (153/555) who did not report household uptake (adjusted PR 2.09, 95% CI: 1.8 to 2.43). Similarly, 57.5% (307/534) of women who reported HIVST among household members self-tested relative to 30.0% (277/922) of women who did not report household uptake (adjusted PR 1.77, 95% CI: 1.56 to 2.01).

There was no evidence that self-rated health status was associated with HIVST (Tables 2 and 3).

## Discussion

The aim of this study was to understand: who is reached by HIVST? We found that self-reported ever HIVST was more prevalent among men than women. Uptake was lowest among adults aged 40 years and older. Women who were married, had children, had higher levels of education, or were wealthier had a higher prevalence of HIVST. Men who reported condomless sex in the past 3 months were also more likely to self-test. Among men and women, the level of previous exposure to HIV testing and household HIVST uptake were associated with HIVST. Given the limited number of population-based studies on HIVST, our study presents a novel evidence on characteristics of individuals likely to self-test in the context of a community-based HIVST program delivered in a high-prevalence, rural African setting.

Men form a disproportionate segment of people unaware of their HIV-positive status. HIVST provides a promising approach for reaching populations unwilling or unable to access facility-based HTS. We found that a higher proportion of men self-tested compared with women, indicating either a higher acceptability of HIVST or greater need for HIV testing. Among men, 28.3% had never tested before the past 12 months compared with 19.1% of women. Furthermore, men who reported condomless sex in the past 3 months had a higher prevalence of HIVST. Ensuring that subgroups with an ongoing risk of HIV infection have access to repeat HIV testing is critical for HIV prevention and could be facilitated by HIVST. In urban Malawi, secondary distribution through sexual partners and community distribution through lay volunteers achieved a high uptake of HIVST in men.^13,14^ Offer of HIVST beyond home-based HTS by community health workers increased knowledge of status by 5% among men in urban Zambia through primary and secondary distribution.^17,28^ We provide supporting evidence on the importance of extending HTS beyond health facilities to improve access and utilization in men and at-risk subgroups.

Our findings show a decreased prevalence of HIVST across higher levels of age group. An earlier study of community-based HIVST in urban Malawi reported similar age patterns, with uptake of HIVST highest in adolescents and lowest in older adults.^13^ A mixed-methods study in Malawi and Zambia found that adolescents and young adults valued HIVST for providing greater autonomy and control over the HIV testing process, including the location and timing of testing and disclosure of results.^29^ An alternative interpretation suggests that adults aged 25–39 years may be less likely to self-test than younger age groups due to availability of HTS through antenatal care. An important subgroup not only routinely accessing facility-based HTS but also less likely to self-test, are adults aged 40 years and older. Despite having the highest HIV prevalence, older adults may not test due to their roles as standard-bearers in their communities and the perception that testing violates sexual decorum.^8,30^ Reported ageism among health care workers might also limit access of HTS.^30^ Ongoing barriers that inhibit utilization of relatively convenient and confidential services need to be understood and addressed.

HIVST was more prevalent among individuals who shared a household with someone who reported HIVST, which may reflect the model of distribution or imply the influence of social relationships on health care utilization. Distributors provided HIVST kits through various approaches, including home-based distribution. As such, uptake by multiple individuals within a household may simply relate to the model of distribution, with preference for home-based distribution of HIVST kits previously described.^29,31^ Alternatively, there is potential for familial networks to influence uptake of HTS through information sharing and support for HIVST and norms setting around HIV prevention behaviors. A social network study found that Tanzanian men were more likely to test for HIV if they had a close friend who also tested, whereas they were less likely to test if they perceived HIV stigma to be present within their social network.^32^

Finally, HIVST was more likely among several subgroups already reached by available HTS. Frequent HIV testing before the past 12 months was strongly associated with increased HIVST. Furthermore, women who reported a higher prevalence of HIVST had similar characteristics to those accessing facility-based HTS, that is, married, more educated, or wealthier women.^33,34^ Uptake among high-coverage and low-risk subgroups can limit the cost-effectiveness of HIVST because community-based programs tend to be more resource and cost-intensive.^35,36^ HIVST programs should therefore consider approaches to maximize complementarity, for example, implementing parallel community sensitization and demand creation activities. The need for community mobilization alongside distribution of HIVST kits is important to meaningfully engage underserved subgroups and build their confidence to access and use HIVST kits.

The main strength of our study is the use of a population-based survey following large-scale, pragmatic implementation of community-based HIVST in a high-prevalence setting. Scale-up of HIVST remains relatively limited in sub-Saharan Africa, with our study providing a unique opportunity to explore uptake of HIVST in the general population. The intervention was delivered through CBDAs, a cadre common throughout Malawi. Our findings are mainly generalizable to similar African settings with equivalent cadres of community volunteers. Furthermore, we used a theoretically informed causal framework to identify and adjust for confounding factors and test for causal associations. Although there are limitations to using observational designs for causal inference, we nevertheless provide important evidence on the characteristics of individuals reached by HIVST. Our study can help to inform provision of differentiated HTS to close remaining gaps in the HIV care cascade.

Our study includes multiple limitations. First, we used a self-reported outcome and exposures of interest, which may be prone to social desirability bias. Second, we may have potential ascertainment bias from nonparticipation. A quarter of eligible men were not available for the end line survey, potentially excluding men with irregular working hours or who migrate for work. Our data on condomless sex in the past 3 months also included a high proportion of missing observations, which we aimed to correct for using multiple imputations. Most models in our analysis, however, did not include the condomless sex measure and were not affected. Third, we used recent condomless sex as a proxy for measuring sexual risk, although it is possible that the reported sexual activity followed HIVST or occurred in stable partnerships. We considered the latter by adjusting our analysis for marital status. Fourth, although we purposefully identified confounding variables using our conceptual framework, we may have some residual confounding. Fifth, we used frequency of HIV testing before the past 12 months to approximate exposure to HIV testing before the community-based HIVST program. Ideally, we would have assessed HIVST among individuals who had not recently tested or were not diagnosed before the program, but our survey did not allow this assessment. Finally, our results are limited to community-based distribution of HIVST kits, with factors associated with HIVST likely to differ by model. Components of our intervention design, including door-to-door implementation, reimbursements for CBDAs, and instructional materials, could influence our findings.

In summary, we analyzed a population-based survey to provide insights into factors associated with ever HIVST within the context of community-based distribution of HIVST kits in rural Malawi. We found that community-based HIVST reached men, younger age groups, and some at-risk individuals. HIVST was also more prevalent among several subgroups already accessing available HTS, including women who were married, more educated, and wealthier. Understanding the characteristics of individuals who are likely to self-test is important to optimize HTS implementation and meet the needs of underserved subgroups. In this study, HIVST was lowest among older adults and individuals with less previous exposure to HIV testing, suggesting the presence of ongoing barriers to HTS. Addressing these barriers, for instance through greater community engagement, will be critical to make the most of this promising strategy.

## Figures and Tables

**Table 1:** Sample Characteristics

	Male (N = 1055)	Female (N = 1456)
	Col % (n)	Col % (n)
Age group		
16–19 yrs	13.1% (138)	14.4% (209)
20–24 yrs	17.3% (183)	18.4% (268)
25–39 yrs	36.2% (382)	36.1% (525)
40+ yrs	33.4% (352)	31.2% (454)
Head of household	60.9% (642)	28.4% (414)
Married or living with partner	73.6% (776)	66.5% (968)
Children	73.7% (778)	86.5% (1260)
Educational attainment		
None	11.4% (120)	22.9% (334)
Primary	68.2% (719)	67.0% (975)
Secondary or higher	20.5% (216)	10.1% (147)
Household wealth status		
Lowest	25.8% (272)	33.7% (490)
Middle	33.6% (354)	32.8% (477)
Highest	40.7% (429)	33.6% (489)
Condomless sex in the past 3 mo*	72.8% (582)	56.5% (608)
Self-rated health status†		
Poor/fair	15.2% (160)	19.2% (279)
Good	56.1% (591)	57.1% (831)
Very good	28.7% (303)	23.7% (345)
No. of HIV tests before the past 12 months‡		
0	28.3% (287)	19.1% (260)
1–2	29.9% (303)	30.7% (418)
3–5	26.6% (270)	33.5% (457)
6+	15.3% (155)	16.7% (228)
Household uptake of HIVST	47.4% (500)	36.7% (534)
Self-tested for HIV	45.0% (475)	40.1% (584)
Tested for HIV	83.4% (880)	89.1% (1298)

The table presents sample characteristics of men and women.

* 26.0% (n = 379) missing for women, 24.2% (n = 255) missing for men.

† 0.07% (n = 1) missing for women, 0.09% (n = 1) missing for men.

‡ 6.4% (n = 93) missing for women, 3.8% (n = 40) missing for men.

**Table 2:** Factors Associated With Ever HIV Self-Testing in Men

		Ever HIV Self-Testing						
	N	Row % (n)	Unadjusted PD % (95% CI)	*P* *	Unadjusted PR (95% CI)	*P* *	Adjusted PR (95% CI)	*P* *
Level 1: Age								
Age group†								
16–19 yrs	138	50.7% (70)	1.2 (−8.3 to 10.8)	< 0.001	1.02 (0.84, 1.22)	< 0.001		
20–24 yrs	183	57.4% (105)	9.6 (1.2 to 17.9)		1.2 (1.02, 1.41)			
25–39 yrs	382	46.9% (179)	0.0		1.0			
40+ yr	352	34.4% (121)	−12.0 (−18.8 to −5.2)		0.74 (0.62, 0.88)			
Level 2a: Sociodemographic factors Head of household								
No	413	48.4% (200)	0.0	0.12	1.0	0.10	1.0	0.88
Yes	642	42.8% (275)	−4.8 (−10.8 to 1.2)		0.9 (0.79–1.02)		0.99 (0.86–1.14)	
Married or living with partner								
No	279	48.7% (136)	0.0	0.07	1.0	0.04	1.0	0.93
Yes	776	43.7% (339)	−6.4 (−13.3 to 0.5)		0.87 (0.76–1.0)		0.99 (0.82–1.20)	
Children								
No	277	48.7% (135)	0.0	0.18	1.0	0.16	1.0	0.09
Yes	778	43.7% (340)	−4.7 (−11.4 to 2.1)		0.91 (0.79–1.04)		1.18 (0.97–1.43)	
Level 2b: Socioeconomic factors Educational attainment‡								
None	120	37.5% (45)	0.0	0.004	1.0	0.007	1.0	0.16
Primary	719	45.6% (328)	11.7 (2.7 to 20.7)		1.29 (1.03–1.61)		1.18 (0.94–1.48)	
Secondary or higher	216	47.2% (102)	18.4 (7.6 to 29.2)		1.52 (1.17–1.97)		1.30 (0.99–1.69)	
Household wealth status								
Lowest	272	42.3% (115)	0.0	0.38	1.0	0.41	1.0	0.16
Middle	354	45.5% (161)	4.3 (−3.3 to 11.9)		1.09 (0.92–1.29)		1.1 (0.93–1.31)	
Highest	429	46.4% (199)	5.1 (−2.4 to 12.5)		1.12 (0.95–1.32)		1.18 (1.0–1.39)	
Level 3a: Sexual behavior factors Condomless sex in the past 3 mo§								
No	218	43.1% (94)	0.0	0.60	1.0	0.67	1.0	0.02
Yes	582	47.9% (279)	2.1 (−5.8 to 9.9)		1.04 (0.87–1.24)		1.37 (1.06–1.76)	
Level 3b: Health behavior factors Self-rated health status								
Poor/fair	160	35.6% (57)	0.0	0.03	1.0	0.06	1.0	0.29
Good	591	46.9% (277)	9.6 (1.6 to 17.5)		1.29 (1.03–1.61)		1.19 (0.95–1.49)	
Very good	303	46.5% (141)	11.3 (2.3 to 20.3)		1.33 (1.04–1.68)		1.20 (0.94–1.53)	
Number of HIV tests before the past 12 mo‖								
0	287	23.3% (67)	0.0	<0.001	1.0	<0.001	1.0	<0.001
1–2	303	47.2% (143)	21.5 (14.3 to 28.7)		1.94 (1.53–2.45)		2.01 (1.59–2.54)	
3–5	270	54.4% (147)	28.1 (20.6 to 35.5)		2.18 (1.73–2.75)		2.29 (1.8–2.9)	
6+	155	61.9% (96)	34.3 (25.7 to 43.0)		2.50 (1.96–3.18)		2.64 (2.06–3.38)	
Household uptake of HIVST								
No	555	27.6% (153)	0.0	<0.001	1.0	<0.001	1.0	<0.001
Yes	500	64.4% (322)	32.2 (26.4 to 38.1)		2.12 (1.82–2.46)		2.09 (1.8–2.43)	

The table presents PDs and PRs for each model. All models account for clustering using a cluster fixed effect. The adjusted set of models account for variables higher in the conceptual framework and associated with the outcome at *P* < 0.10 level. Models in Level 1 adjusted for cluster. Models in Level 2 adjusted for cluster and age. Models in Level 3 adjusted for cluster, sociodemographic variables, and socioeconomic variables.

* *P* value for the Wald test.

† The 25–39-year age group was used as the base category due to a higher HIV testing prevalence in this subgroup. Test for linear trend, *P* < 0.001.

‡ Test for linear trend, *P* = 0.26.

§ Results of multiple imputation analysis presented. Complete case analysis, PD: 3.3% (24.2%, 10.9%), *P* = 0.39; PR: 1.06 (0.9–1.25), *P* = 0.49; adjusted PR: 1.33 (1.05–1.68), *P* = 0.02.

‖ Test for linear trend, *P* = 0.003.

PD, prevalence difference.

**Table 3:** Factors Associated With Ever HIV Self-Testing in Women

		Ever HIV Self-Testing						
	N	Row % (n)	Unadjusted PD % (95% CI)	*P* *	Unadjusted PR (95% CI)	*P* *	Adjusted PR (95% CI)	*P* *
Level 1: Age								
Age group†								
16–19 yrs	209	46.4 (97)	4.9 (−2.7 to 12.5)	<0.001	1.12 (0.93 to 1.33)	<0.001		
20–24 yrs	268	50.0 (134)	6.8 (0.0 to 14.0)		1.15 (0.99 to 1.34)			
25–39 yrs	525	41.3 (217)	0.0		1.0			
40+ yrs	454	30.0 (136)	−11.8 (−17.6 to −6.0)		0.71 (0.6 to 0.84)			
Level 2a: sociodemographic factors Head of household								
No	1042	42.3 (441)	0.0	0.007	1.0	0.01	1.0	0.39
Yes	414	34.5 (143)	−7.4 (−12.8 to −2.0)		0.83 (0.72 to 0.96)		0.94 (0.8 to 1.09)	
Married or living with partner								
No	488	32.8 (160)	0.0	<0.001	1.0	<0.001	1.0	0.002
Yes	968	43.8 (424)	10.9 (5.7 to 16.0)		1.31 (1.14 to 1.51)		1.26 (1.09 to 1.46)	
Children								
No	196	37.8 (74)	0.0	0.65	1.0	0.59	1.0	0.01
Yes	1260	40.5 (510)	1.6 (−5.3 to 8.5)		1.05 (0.87 to 1.28)		1.38 (1.08 to 1.76)	
Level 2b: Socioeconomic factors Educational attainment‡								
None	334	30.5 (102)	0.0	<0.001	1.0	<0.001	1.0	<0.001
Primary	975	41.9 (409)	13.5 (8.0 to 19.0)		1.47 (1.24 to 1.75)		1.31 (1.09 to 1.57)	
Secondary or higher	147	49.7 (73)	25.2 (15.7 to 34.7)		1.99 (1.57 to 2.52)		1.66 (1.29 to 2.13)	
Household wealth status								
Lowest	490	35.7 (175)	0.0	0.003	1.0	0.002	1.0	0.002
Middle	477	39.8 (190)	4.0 (−1.9 to 9.9)		1.11 (0.94 to 1.3)		1.08 (0.92 to 1.26)	
Highest	489	44.8 (219)	10.6 (4.5 to 16.7)		1.31 (1.13 to 1.53)		1.3 (1.12 to 1.52)	
Level 3a: Sexual behavior factors Condomless sex in the past 3 mo§								
No	469	31.1 (146)	0.0	<0.001	1.0	<0.001	1.0	0.19
Yes	608	45.9 (279)	12.6 (7.3 to 17.9)		1.38 (1.19 to 1.6)		1.21 (0.91 to 1.61)	
Level 3b: Health behavior factors Self-rated health status								
Poor/fair	279	33.0 (92)	0.0	0.005	1.0	0.008	1.0	0.29
Good	831	41.3 (343)	8.6 (2.4 to 14.8)		1.26 (1.05 to 1.51)		1.08 (0.9 to 1.29)	
Very good							
No. of HIV tests before the past 12 mo‖	345	42.9 (148)	11.8 (4.3 to 19.3)		1.37 (1.12 to 1.68)		1.17 (0.95 to 1.44)	
0	260	18.1 (47)	0.0	<0.001	1.0	<0.001	1.0	<0.001
1–2	418	39.2 (164)	18.9 (12.6 to 25.2)		2.13 (1.6 to 2.83)		2.03 (1.52 to 2.71)	
3–5	457	48.1 (220)	26.0 (19.6 to 32.4)		2.49 (1.89 to 3.29)		2.51 (1.89 to 3.34)	
6+	228	48.7 (111)	27.3 (19.2 to 35.3)		2.57 (1.92 to 3.43)		2.59 (1.91 to 3.51)	
Household uptake of HIVST								
No	922	30.0 (277)	0.0	<0.001	1.0	<0.001	1.0	<0.001
Yes	534	57.5 (307)	24.4 (19.3 to 29.6)		1.79 (1.58 to 2.02)		1.77 (1.56 to 2.01)	

The table presents PDs and PRs for each model. All models account for clustering using a cluster fixed effect. The adjusted set of models account for variables higher in the conceptual framework and associated with the outcome at *P* < 0.10 level. Models in Level 1 adjusted for cluster. Models in Level 2 adjusted for cluster and age. Models in Level 3 adjusted for cluster, sociodemographic variables, and socioeconomic variables.

* *P* value for the Wald test.

† The 25–39-year age group was used as the base category due to a higher HIV testing prevalence in this subgroup. Test for linear trend, *P* < 0.001.

‡ Test for linear trend, *P* = 0.01.

§ Results of multiple imputation analysis presented. Complete case analysis, PD: 13.8 (8.3, 19.4), *P* < 0.001; PR: 1.44 (1.23–1.68), *P* < 0.001; adjusted PR: 1.24 (0.94 –1.64), *P* = 0.13.

‖ Test for linear trend, *P* = 0.002.

PD, prevalence difference.
